# Synthesis and Biological Activity of Some New 1,3,4-Thiadiazole and 1,2,4-Triazole Compounds Containing a Phenylalanine Moiety

**DOI:** 10.3390/molecules14072621

**Published:** 2009-07-16

**Authors:** Mihaela Moise, Valeriu Sunel, Lenuta Profire, Marcel Popa, Jacques Desbrieres, Cristian Peptu

**Affiliations:** 1 Department of Organic Chemistry and Biochemistry, Faculty of Chemistry, “Al. I. Cuza” University, Iasi, 11 B-dul Carol I, 700506, Romania; E-mails: moise_mihaela82@yahoo.com (M.M.), vsunel@uaic.ro (V.S.); 2 Department of Pharmaceutical Chemistry, Faculty of Pharmacy, “Gr. T. Popa” Medicine and Pharmacy University, 16 Universitatii Street, 700115, Romania; 3 Department of Natural and Synthetic Polymers, Faculty of Chemical Engineering and Environmental Protection, “Gh. Asachi” Technical University, 71A B-dul Mangeron, Iasi, 700050, Romania; E-mail: marpopa@ch.tuiasi.ro (M.P.); 4 Pau et Pays de l’Adour University, Institut Pluridisciplinaire de Recherche sur l’Environnement et les Matériaux – Equipe de Physique et Chimie des Polymèrs (IPREM/EPCP), UMR 5254 CNRS, Hélioparc Pau Pyrénées 2, av. President Angot, 64053 Pau Cedex 09, France; E-mail: jacques_desbrieres@univ-pau.fr (J.D.); 5 “Petru Poni" Institute of Macromolecular Chemistry, 41A Grigore Ghica Voda Alley 700487 Iasi, Romania

**Keywords:** phenylalanine, thiosemicarbazide, 1,3,4-thiadiazole, 1,2,4-triazole, anti-inflammatory activity

## Abstract

New 1,3,4-thiadiazole, **6, 7** and 1,2,4-triazole derivatives, **8, 9** containing a phenylalanine moiety have been synthesized by intramolecular cyclization of 1,4-disubstituted thiosemicarbazides, **4, 5**, in acid and alkaline media, respectively; the thiosemicarbazides were obtained by reaction of hydrazide **3** with appropriate aromatic isothiocyanates. The toxicity of the synthesized compounds was evaluated and the anti-inflammatory study of the triazole compound **9** established an appreciable anti-inflammatory activity that is comparable with that of other nonsteroidal anti-inflammatory agents.

## 1. Introduction

The progress achieved in the synthesis of heterocyclic compounds with biological potential is due to improvement of the methodological study of tested substances too. It is known that many 1,3,4-thiadiazole and 1,2,4-triazole derivatives have biological activity, with their antibacterial [1,2,3], antimycobacterial [4,5], antimycotic [6], antifungal [7,8], antidepressive [9], and cardiotonic [10] action being notable. Recent research has also established for these heterocycles an analgesic [11] and anti-inflammatory [12,13] activity. Meanwhile, *N*-acylated amino acids are known for their hepatoprotective [14], antimicrobial [15,16] and antitumoral [17,18] action. Taking these data into account, in the present study, some new 1,3,4-thiadiazole and 1,2,4-triazole derivatives having a phenylalanine moiety have been synthesized and their structure confirmed by elemental and spectral (FT-IR, ^1^H-NMR, MS) analyses. The degree of toxicity of the compounds was established and the potential anti-inflammatory activity of the triazole compound **9** was also investigated. 

## 2. Results and Discussion

### 2.1. Chemistry

**Scheme 1:**
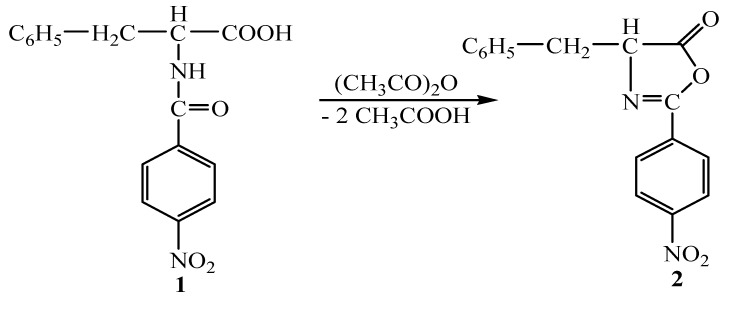
Synthesis of oxazolone **2.**

The synthesis of new 1,3,4-thiadiazole and 1,2,4-triazole compounds was performed in several steps. In the first step, 2-(*p*-nitrophenyl)-4-benzyl-Δ^2^-oxazolin-5-one (**2**) was obtained by heating *N*-(*p*-nitrobenzoyl)-L-phenylalanine (**1**) at 70-75°C for 60 minutes under the dehydrating action of excess acetic anhydride (1:10 molar rate) (Scheme 1).

The structure of oxazolone **2** was established through by spectroscopic (IR, ^1^H-NMR, MS) as well as elemental analyses data. In the IR spectra the absorption bands characteristic for the >C=O lactonic group and >C=N bond were identified at 1,825 cm^-1^ and 1,620 cm^-1^, respectively, and in the ^1^H-NMR spectrum the proton signals due to the heterocyclic proton appeared at 4.85 ppm. In the MS spectra the base peak was observed at m/z = 297.09 and it was assigned as protonated compound having the monoisotopic mass 296.09. The [M+Na]^+^ species at m/z = 319.07 and Na adducts of two oxazolone molecules were also detected. The accurate mass measurements allowed the evaluation of the isotopic profile for the peaks situated at 297.09, 298.09 and 299.09.

**Scheme 2:**
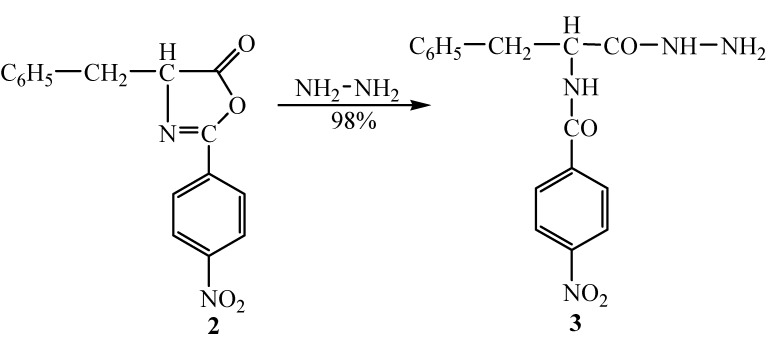
Synthesis of hydrazide **3.**

By reaction of Δ^2^-oxazolin-5-one **2** with hydrazine hydrate (98% solution) in anhydrous dioxane under reflux for 3 hours, *N*-(*p*-nitrobenzoyl)-D,L-phenylalanine hydrazide (**3**) was obtained (Scheme 2).

The structure of hydrazide **3** was established through by spectroscopic (IR, ^1^H-NMR) as well as elemental analyses data. The IR spectra showed two characteristic bands for NH groups at 2,857 cm^-1^ and 3,269 cm^-1^, respectively, and the absorption band for CO amide group appeared at 1,661 cm^-1^. In the ^1^H-NMR spectrum the NH groups appeared as two signals at 9.0 ppm and 10.40 ppm, while the proton signals of the NH_2_ group were identified at 5.37 ppm.

**Scheme 3:**
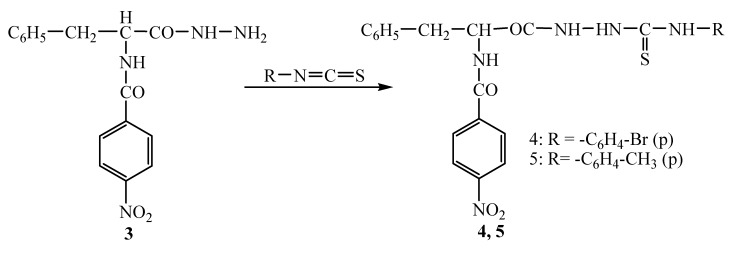
Synthesis of thiosemicarbazides **4, 5.**

The new 1,4-disubstituted thiosemicarbazides **4, 5** were obtained through reaction of hydrazide **3** with *p*-bromophenyl- and *p*-tolylisothiocyanates in methanol media under reflux (Scheme 3).

The formation of thiosemicarbazides **4, 5** was indicated in the IR spectra by a shift in the CO amide group absorption of hydrazide **3** from 1,661 cm^-1^ to 1,654-1,683 cm^-1^ in the thiosemicarbazide derivatives and by the band of >C=S bond that appears at 1,234-1,299 cm^-1^. In the ^1^H-NMR spectra the protons linked to nitrogen appear at 9.00-10.48 ppm. In the MS spectra of thiosemicarbazide **4** the base peak was observed at m/z = 564.02 and it was assigned as its Na adduct (564 = 541 +23). Beside the Na adduct there were observed (M+H)^+^ and (M+K)^+^ adducts at m/z = 542 and 580, respectively. Two cluster molecules charged with Na were also observed at m/z = 1105 (541×2 + 23). For the MS spectra of thiosemicarbazide **5** the base peak was observed at m/z = 500.13 and it is associated with the presence of the Na adduct of this thiosemicarbazide (477+23). The peaks accompanying the base peak are (M+H)^+^ at m/z = 478 and (M+K)^+^ at 516. An additional peak at m/z = 977 representing the Na adduct of two thiosemicarbazide molecules (977=477×2+23) was also observed. 

**Scheme 4:**
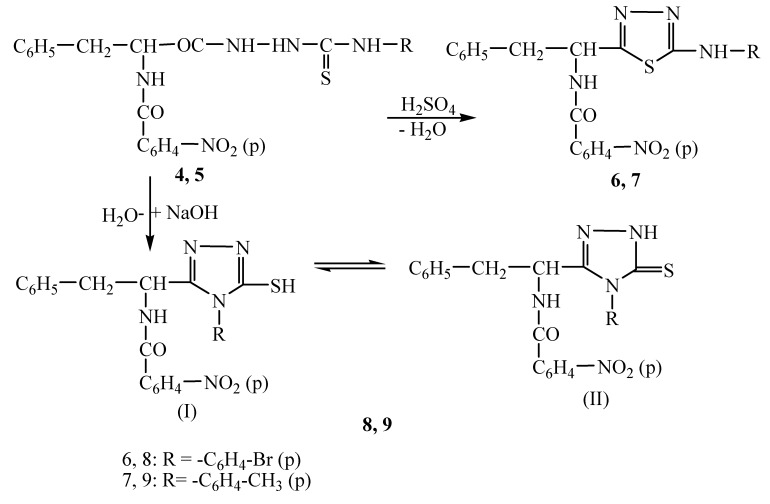
Synthesis of 1,3,4-thiadiazole (**6, 7**) and 1,2,4-triazole (**8, 9**) compounds.

In the last step by intramolecular cyclization of thiosemicarbazides **4, 5** in acid and alkaline media, respectively, new 1,3,4-thiadiazole and 1,2,4-triazole compounds **6, 7** and **8, 9** were obtained (Scheme 4). The 1,2,4-triazole derivatives can exist in two tautomeric forms – thiole (I) and thione (II), the reaction conditions leading to the chemical stabilization of the tautomeric thione form [19,20].

The structure of the final compounds was confirmed by elemental and spectral (FT-IR, ^1^H-NMR and MS) analyses. In the IR spectra of the 1,3,4-thiadiazoles **6, 7** the >C=N group appears as an absorption band at 1,665-1,672 cm^-1^, and the C-S bond was identified at 640-652 cm^-1^. In the ^1^H-NMR spectra of 1,3,4-thiadiazoles **6, 7** the proton signal for the amine group appears as singlet at 6.00-6.05 ppm and that for the amide group appears at 9.08-9.10 ppm. The CH_3_ group is present as a singlet at 2.34 ppm. In the mass spectrum of thiadiazole **6** the base peak was observed at m/z = 524, corresponding to the [M-H]^+^ ions of the product. The base peak for thiadiazole **7** is situated at m/z = 460.13 corresponding to the [M-H]^+^ ions of the product.

In the IR spectra of 1,2,4-triazoles **8, 9** the >C=N group was identified at 1,566-1,595 cm^-1^ and the characteristic C=S function absorption band appeared at 1,300-1,320 cm^-1^ region. In the ^1^H-NMR spectra the proton signal for the NH from the triazole ring was observed as a singlet at 12.80 ppm. The mass spectra of triazole **8** showed the base peak at m/z = 524.03 corresponding to the [M-H]^+^ ions of the product with monoisotopic mass of 523.03 g/mol. The smaller peaks are assigned as Na and K adducts of the same molecule or species adducts formed by physical association of two molecules (e.g. 1069.01 = 2×523+23). The base peak observed in the case of triazole **9** is situated at m/z = 460.13 corresponding to the [M-H]^+^ ion of the product. 

### 2.2. Biological Activities

The synthesized compounds were investigated for their toxicity (Table 1). It was ascertained that all tested compounds have low toxicity, the lowest toxic compounds being 1,3,4-thiadiazole **7** and 1,2,4-thiazole **9**, both having a *p*-tolyl moiety in the structure.

**Table 1:** DL50 values of the tested compounds.

Comp.	DL_50_ (mg/kg body weight)			
	24 hours	48 hours	7 days	Average
**2**	1,110	1,110	975	1,065
**3**	1,100	1,100	875	1,025
**4**	1,650	1,650	1,560	1,620
**5**	2,050	2,050	1,810	1,970
**6**	2,350	2,350	2,110	2,270
**7**	5,200	5,200	3,460	4,620
**8**	2,250	2,250	1,950	2,150
**9**	5,150	5,150	4,730	5,010

The potential anti-inflammatory activity of the 1,2,4-triazole (**9**) comparative with standard nonsteroidal anti-inflammatory drugs (indometacine, phenylbutazone, acetylsalicylic acid) was also investigated (Table 2).

The substances were administered in two concentrations, 1/10 of DL_50_ and 1/5 of DL_50_, respectively, relative to body weight of the animal tested. From the results presented in Table 2 it is observed that at small doses (1/10 DL_50_) only indometacine has an appreciable anti-inflammatory activity (60.30% inhibition of oedema) while at higher doses (1/5 DL_50_) all tested compounds reduced the nystatin-induced oedema (45.33-61.20% inhibition of oedema). The activity of the 1,2,4-triazole (**9**) is similar to the phenylbutazone’s activity (49.03% inhibition of oedema). This effect could be explained through both the lysosomal stabilization and the inhibition of prostaglandin biosynthesis [21,22,23].

**Table 2:** The anti-inflammatory effects of tested compounds.

Comp.	Dose (mg/kg body, p.o.)	Inflammatory oedema (mL/100 g body)		Inhibition (%)
		Control	Comp.	
**Indometacine**	5*	0.280±0.110	0.111±0.008	60.30
	10**	0.310±0.005	0.124±0.070	61.20
**Phenylbutazone**	50*	0.280±0.110	0.190±0.062	32.10
	100**	0.310±0.005	0.158±0.025	49.03
**Acetylsalicylic Acid**	150 *	0.310±0.005	0.250±0.057	19.35
	250**	0.300±0.100	0.164±0.072	45.33
**1,2,4-Triazole (9)**	80*	0.280±0.110	0.186± 0.060	33.57
	160**	0.310±0.095	0.158±0.024	49.03

* = 1/10 DL_50_, **1/5DL_50_

## 3. Experimental Section

### 3.1. Chemistry

#### 3.1.1. General

All melting points were determined on a Melt-Temp R apparatus equipped with a digital thermometer and are uncorrected. The combustion analysis was performed on an Elemental Exeter Analytical CE 440 Apparatus. The IR spectra were measured as potassium bromide pellets on a Digilab Scimitar Series FT-IR Spectrophotometer; the wave numbers are given in cm^-1^. The ^1^H-NMR spectra were recorded in DMSO-d_6_ or CD_3_COCD_3_ solutions on Bruker ARX-300 spectrometer at ambient temperature. Chemical shifts were recorded as δ values in parts per millions (ppm) and were indirectly referenced to tetramethylsilane via residual solvent signal (2.49 for ^1^H). MS spectra were obtained using an instrument produced by Agilent Technologies, Wilmington, DE, USA. The instrument, Accurate Mass Q-TOF LC/MS 6520 was operated via the manufacture’s software, Mass Hunter. Samples were dissolved in acetonitrile/water mixture (95/5 v/v) to obtain a concentration of 10 μg/mL and 0.05 mL were directly injected into the electrospray source using the autosampler at a rate of 0.05 mL/min. The instrument was operated in High resolution mode with an acquisition rate of 4 GHz. The source voltage was set at 4,000 V, the spray gas flow at 5 L/min, heating gas temperature at 325 °C and the fragmentor potential at 215 V. All chemical reagents were obtained from the Aldrich Chemical Company.

#### 3.1.2. Synthesis of 2-(p-nitrophenyl)-4-benzyl-Δ^2^-oxazolin-5-one (**2**)

*N*-(*p*-nitrobenzoyl)-L-phenylalanine (5.32 g, 0.018 mol) was dissolved in acetic anhydride (18 mL) and heated under reflux at 70-75ºC for 45-60 minutes. After cooling, the solution was added under stirring over a mixture of dried petroleum ether (55 mL) and dried ethyl ether (35 mL). The reaction mixture was stirred for 15 minutes, the ether layer was removed and the oily product obtained was washed many times with dried ethyl ether until a solid product was obtained that was filtered and dried for 8-10 hours under vacuum at 40-45 °C. Crystallization from anhydrous dioxane gave a pure yellow solid (383 mg, 72% yield), mp 120-123 °C; IR (cm^-1^) v: 1,825 (CO), 1,620 (C=N), 1,520 (asymmetric NO_2_ vibrations), 1,342 (symmetric NO_2_ vibrations), 786 (aromatic CH); ^1^H-NMR (CD_3_COCD_3_) δ: 3.15 (m, 1H, CH_2_), 3.25 (m, 1H, CH_2_), 4.85 (t, 1H, CH), 7.20 (m, 5H, Ph), 8.15 (d, 2H, C_6_H_4_), 8.35 (d, 2H, C_6_H_4_); MS (acetonitrile/water 95/5, v/v) m/z: 297.09 (base peak), 319.07 (14.5%), 615.11 (21%); Anal. calcd. for C_16_H_12_N_2_O_4_ (%): C, 64.86; H, 4.05; N, 9.45; found: C, 65.32; H, 4.26; N, 9.91.

#### 3.1.3. N-(p-nitrobenzoyl)-D,L-phenylalanine hydrazide (3)

2-(*p*-Nitrophenyl)-4-benzyl-Δ^2^-oxazolin-5-one (**2,** 5.94 g, 0.02 mol) was dissolved in dioxane (50 mL) and hydrazine hydrate solution (98%, 0.5 mL, 0.02 mol) was added. The reaction mixture was heated at 65-70ºC for three hours, under reflux on a thermostated silicone oil bath. The solvent was removed by distillation under reduced pressure till 10-15 mL, and then water was added until a yellow semisolid product was separated. Crystallization from ethanol-water mixture gave a pure yellow solid (514 mg, 78.35% yield), mp 210-212 °C; IR (cm^-1^) v: 3,269, 2,857 (NH), 1,661 (CO), 1,526 (asymmetric NO_2_ vibrations), 1,346 (symmetric NO_2_ vibrations), 723, 868 (aromatic CH); ^1^H-NMR (DMSO-d_6_, 300 MHz) δ: 3.30 (m, 2H, CH_2_), 4.80 (m, 1H, CH), 5.37 (s, 2H, NH_2_), 7.40 (m, 5H, Ph), 8.05 (d, 2H, C_6_H_4_), 8.30 (d, 2H, C_6_H_4_), 9.0 (m, 1H, NH), 10.40 (s, 1H, NH); Anal. calcd. for C_16_H_16_N_4_O_4_ (%): C, 58.53; H, 4.87; N, 17.07; found: C, 58.75; H, 5.33; N, 17.46. 

#### 3.1.4. General procedure for synthesis of 1,4-disubstituted thiosemicarbazides **4, 5**

*N*-(*p*-nitrobenzoyl)-D,L-phenylalanine hydrazide (**3**, 1.64 g, 0.005 mol) was dissolved in dried methanol (10 mL) and a solution of the corresponding isothiocyanate (0.005 mol) in dried methanol (10 mL) was added. The reaction mixture was heated under reflux at 70-80ºC for three hours. After cooling the solvent was evaporated under reduced pressure and the solid was dried under vacuum at room temperature. The rough product was purified by crystallization from ethanol. 

*1-[N-(p-nitrobenzoyl)-D,L-phenylalanyl-4-(p-bromophenyl)-thiosemicarbazide* (**4**): white solid, 184 mg (68.33% yield); mp 188-190°C; IR (cm^-1^) v: 3,541, 3,460, 3,169 (NH), 1,683 (CO), 1,573 (asymmetric NO_2_ vibrations), 1,379 (symmetric NO_2_ vibrations), 1,299 (C=S), 754, 796 (aromatic CH), 565 (C-Br); ^1^H-NMR (DMSO-d_6_, 300 MHz) δ: 3.04 (m, 1H, CH_2_), 3.10 (m, 1H, CH_2_), 4.70 (m, 1H, CH), 7.17-7.33 (m, 6H, Ar CH), 7.56 (m, 3H, Ar CH), 8.01 (d, 2H, Ar CH), 8.31 (m, 2H, Ar CH), 9.0 (d, 1H, NH), 9.91 (s, 1H, NH), 10.48 (s, 1H, NH); MS (acetonitrile/water 95/5, v/v) m/z: 564 (base peak) (541+23), 542 (541+1) (30%), 580 (541+39) (19%), 1105 (541×2+23) (4%); Anal. calcd. for C_23_H_20_N_5_O_4_BrS (%): C, 51.01; H, 3.69; N, 12.93; Br, 14.78; S, 5.91; found: C, 51.37; H, 3.88; N, 13.35; Br, 15.19; S, 6.27. 

*1-[N-(p-nitrobenzoyl)-D,L-phenylalanyl-4-(p-tolyl)-thiosemicarbazide* (**5**): yellow solid, 186 mg (78.35% yield), mp 179-180°C; IR (cm^-1^) v: 3,321, 3,211 (NH), 1,654 (CO), 1558 (asymmetric NO_2_ vibrations), 1,357 (symmetric NO_2_ vibrations), 1,234 (C=S), 873 (aromatic CH); ^1^H-NMR (DMSO-d_6_, 300 MHz) δ: 2.29 (s, 3H, CH_3_), 3.40 (m, 1H, CH_2_), 4.70 (m, 1H, CH), 7.14-7.50 (m, 8H, Ar CH), 7.95 (s, 1H, Ar CH), 8.01 (d, 2H, Ar CH), 8.30 (d, 2H, Ar CH), 9.19 (s, 2H, NH), 9.71 (s, 1H, NH), 10.44 (s, 1H, NH); MS (acetonitrile/water 95/5, v/v) m/z: 500.13 (base peak) (477+23), 478.15 (477+1) (39.5%), 516.11 (477+39) (13%), 977 (477×2+23) (30%). Anal. calcd. for C_24_H_23_N_5_O_4_S (%): C, 60.37; H, 4.82; N, 14.67; S, 6.70; found: C, 60.81; H, 5.01; N, 15.10; S, 7.19.

#### 3.1.5. General procedure for synthesis of 1,3,4-thiadiazoles **6, 7**

To the corresponding thiosemicarbazide **4, 5 ** (0.006 mol), concentrated H_2_SO_4_ (1 mL) was added under stirring. The reaction mixture was stirred at room temperature for one hour and then was added dropwise in cold water and stirred again till a solid product was obtained that was separated and dried under vacuum at 45-50ºC. The rough product was purified by crystallization from ethanol. 

*2-*[1-(p-Nitrobenzoylamino)-2-phenyl]*-ethyl-5-(p-bromophenylamino)-1,3,4-thiadiazole* (**6**): light yellow solid, 223 mg (71.50% yield), mp 151-153°C; IR (cm^-1^) v: 3,263, 3,066 (NH), 1,672 (C=N), 1,620 (CO), 1,520 (asymmetric NO_2_ vibrations), 1,341 (symmetric NO_2_ vibrations), 812 (aromatic CH), 750 (C-Br), 640 (C-S); ^1^H-NMR (DMSO-d_6_, 300 MHz) δ: 2.60 – 2.65 (m, 2 H, CH_2_), 4.76 (m, 1H, NH), 6.00 (m, 1H, CH), 6.56 (d, 2H, ArCH), 7.63 – 7.78 (m, 5H, ArCH), 8.30 (d, 2H, ArCH), 8.40 (d, 2H, ArCH), 8.80 (d, 2H, ArCH), 9.10 (s, 1H, -NH-CO-); MS (acetonitrile/water 95/5, v/v) m/z: 524 (base peak), 1047 (1.5%); Anal. calcd. for C_23_H_18_N_5_O_3_BrS (%): C, 52.67; H, 3.43; N, 13.35; Br, 15.26; S, 6.10; found: C, 53.02; H, 3.89; N, 13.84; Br, 15.67; S, 6.58.

*2-*[1-(p-Nitrobenzoylamino)-2-phenyl]*-ethyl-5-(p-tolylamino)-1,3,4-thiadiazole* (**7**): yellow solid, 195 mg (71.27% yield), mp 180-182°C; IR (cm^-1^) v: 3,259, 3,192 (NH), 1,665 (C=N), 1,620 (CO), 1,552 (asymmetric NO_2_ vibrations), 1,384 (symmetric NO_2_ vibrations), 815 (aromatic CH), 652 (C-S); ^1^H-NMR (DMSO-d_6_, 300 MHz) δ: 2.34 (s, 3H, CH_3_), 2.60 – 2.63 (m, 2H, CH_2_), 4.76 (m, 1H, NH), 6.05 (m, 1H, CH), 6.58 (d, 2H, ArCH), 7.63-7.80 (m, 5H, ArCH), 8.33 (d, 2H, ArCH), 8.45 (d, 2H, ArCH), 8.75 (d, 2H, ArCH), 9.08 (s, 1H, -NH-CO-); MS (acetonitrile/water 95/5, v/v) m/z: 460.13 (base peak), 482.02 (1%), 919.14 (2%), 941.12 (1%); Anal. calcd. for C_24_H_21_N_5_O_3_S (%): C, 62.74; H, 4.57; N, 15.25; S, 6.97; found: C, 63.22; H, 4.91; N, 15.70; S, 7.47.

#### 3.1.6. General procedure for the synthesis of 1,2,4-triazoles **8, 9**

To corresponding thiosemicarbazide **6, 7** (0.0014 mol), a solution of NaOH 2N (10 mL) was added. The reaction mixture was heated under reflux at 80-90 °C for four hours and then a solution of HCl 1N was added until it reached pH 4.5 when a solid product was formed. The rough product was separated and dried under vacuum at 55-60ºC and then it was recrystallized from ethanol. 

*4-(p-bromophenyl)-5-*[1-(p-nitrobenzoylamino)-2-phenyl-ethyl]*-3-thio-1,2,4-triazole* (**8**): yellow-orange solid, 46 mg (64.45% yield), mp 147-149°C; IR (cm^-1^) v: 3,062 (NH), 1,664 (CO), 1,595 (C=N), 1,546 (asymmetric NO_2_ vibrations), 1,300 (C=S), 1,332 (symmetric NO_2 _ vibrations), 925, 873 (aromatic CH), 752 (C-Br); ^1^H-NMR (DMSO-d_6_, 300 MHz) δ: 2.62 – 2.66 (m, 2H, CH_2_), 4.86 (m, 1H, CH), 7.25 – 7.30 (m, 5H, ArCH), 7.80 (d, 2H, ArCH), 8.39 ( d, 2H, ArCH), 8.50 (d, 2H, ArCH), 8.75 (d, 2H, ArCH), 9.05 (m, 1H, -NH-CO-), 12.80 (s, 1H, NH); MS (acetonitrile/water 95/5, v/v) m/z: 524.03 (base peak), 541.94 (33%), 563.92 (25%), 1069.01 (9%); Anal. calcd. for C_23_H_18_N_5_O_3_BrS (%): C, 52.67; H, 3.43; N, 13.35; Br, 15.26; S, 6.10; found: C, 53.08; H, 3.85; N, 13.85; Br, 15.26; S, 6.49.

*5-*[1-(p-nitrobenzoylamino)-2-phenyl-ethyl]*-3-thio-4-(p-tolyl)-1,2,4-triazole* (**9**): brown solid, 43 mg (69% yield), mp 172-174°C; IR (cm^-1^) v: 3,437 (NH), 1,670 (CO), 1,566 (C=N), 1,520 (asymmetric NO_2_ vibrations), 1,320 (C=S), 1,338 (symmetric NO_2 _ vibrations), 962, 785 (aromatic CH); ^1^H-NMR (DMSO-d_6_, 300 MHz) δ: 2,32 (t, 3H, CH_3_), 2.57 – 3.35 (m, 2H, CH_2_), 7.14 – 7.19 (m, 4H, ArCH), 7.78 – 7.82 (m, 5 H, ArCH), 8.45 (d, 2H, ArCH), 8.75 (d, 2H, ArCH), 9.08 (s, 1H, -NH-CO-), 12.80 (s, 1H, NH); MS (acetonitrile/water 95/5, v/v) m/z: 460.13 (base peak), 482.04 (18%), 941.13 (11%). Anal. calcd. for C_24_H_21_N_5_O_3_S (%): C, 62.74; H, 4.57; N, 15.25; S, 6.97; found: C, 63.16; H, 5.06; N, 15.62; S, 7.41.

### 3.2. Toxicity study

The acute toxicity was estimated by intraperitoneal administration of the compounds **2-9** as a suspension in Tween 80 to groups of six male mice, each weighing 20-22 g, according to the classical laboratory methodology [24]. The animals were monitored and the death rate ascertained after 24 hours, 48 hours and 7 days. The DL_50_ was established using the Spearman-Karber method [25].

### 3.3. Anti-inflammatoty study

The nystatin-induced paw oedema in rats was realized according to the Niemegeers *et al*. method [24]. A group of six rats weighing 120-160 g housed at 21.5 °C with free-food and water for 24 hours was used. After the measurement of the initial volume of paw according to the Whitehouse method [19], the animals were injected in their left posterior paw with 0.1 mL nystatin suspension (3,500 U/mg) with concentration of 65 mg/mL in 0.9% NaCl solution. Two hours after injection, the triazole compound **9** and the standard anti-inflammatory drugs (indometacine, phenylbutazone and acetylsalicylic acid) were administered through tube-feeding, in 1/10 and 1/5 of DL_50_ dose as a suspension in CMC 0.5%, 20 ml/kg body. The control groups received only vehicle. The final volume of each paw was measured four hours later, and the difference between the initial and the final paw volume was established. This difference, expressed in mL/100 g body, represents the inflammatory oedema; the mean paw volume in the drug-treated and control groups respectively was also established. The percentage oedema was calculated, using student’s – t test for statistic interpretation. 

## 4. Conclusions

New 1,3,4-thiadiazole (**6-7**) and 1,2,4-triazole (**8-9**) compounds having a D,L-phenylalanine moiety were synthesized by intramolecular cyclization of 1,4-disubstituted thiosemicarbazide in acid and alkaline medium, respectively. The corresponding thiosemicarbazides (**4, 5**) were obtained by addition of *N*-(*p*-nitrobenzoyl)-D,L-phenylalanine hydrazide to the aromatic isothiocyanates. The DL_50_ values for all synthesized compounds were established, all compounds having a low toxicity. The potential anti-inflammatory effects of the thiazole **9** by using the nystatin-induced paw oedema in rats was also studied. The triazole **9**, at a dose of 160 mg/kg body (1/5 DL_50_), reduced the inflammatory oedema considerably, this action being comparable with that of the tested standard anti-inflammatory drugs.
